# Preparation and characterization of antibody-drug conjugates acting on HER2-positive cancer cells

**DOI:** 10.1371/journal.pone.0239813

**Published:** 2020-09-28

**Authors:** Zu-Chian Chiang, Yi-Kai Chiu, Cheng-Chung Lee, Nai-Shu Hsu, Yueh-Liang Tsou, Hong-Sen Chen, Horng-Ru Hsu, Tzung-Jie Yang, An-Suei Yang, Andrew H. -J. Wang

**Affiliations:** 1 Institute of Biological Chemistry, Academia Sinica, Taipei, Taiwan; 2 Genomics Research Center, Academia Sinica, Taipei, Taiwan; 3 Drug Metabolism & Pharmacokinetics, Institute for Drug Evaluation Platform, Development Center for Biotechnology, Taipei, Taiwan; Duke University School of Medicine, UNITED STATES

## Abstract

Two systems of antibody-drug conjugates (ADCs), noncleavable H32-DM1 and cleavable H32-VCMMAE, were developed by using different linkers and drugs attached to the anti-HER2 antibody H32, which is capable of cell internalization. Activated functional groups, including an N-hydroxysuccinimidyl (NHS) ester and a maleimide, were utilized to make the ADCs. Mass spectrometry, hydrophobic interaction chromatography, polyacrylamide gel electrophoresis, and *in vitro* cell assays were performed to analyze and optimize the ADCs. Several H32-VCMMAE ADCs were established with higher DARs and greater synthetic yields without compromising potency. The anticancer efficacy of H32-DM1 was 2- to 8-fold greater than that of Kadcyla^®^. The efficacy of H32-VCMMAE was in turn better than that of H32-DM1. The anticancer efficacy of these ADCs against N87, SK-BR-3 and BT474 cells was in the following order: H32-VCMMAE series > H32-DM1 series > Kadcyla^®^. The optimal DAR for H32-VCMMAE was found to be 6.6, with desirable attributes including good cell penetration, a releasable payload in cancer cells, and high potency. Our results demonstrated the potential of H32-VCMMAE as a good ADC candidate.

## Introduction

According to the World Health Organization, cancer was the second leading cause of death globally in 2018 [1, 2]. Therefore, cancer therapy is important for the improvement of health. Targeted therapy is a new trend in cancer treatment [3]. Antibody-drug conjugates (ADCs) are potentially both highly specific and effective for targeted cancer therapy [4, 5]. The antibody portion of ADCs is preferably able to enter cancer cells, and the conjugated drug can be released to kill the cancer cells. Currently, T-DM1 is the only commercial ADC among HER2-targeted drugs, and is a noncleavable conjugated drug system. However, a cleavable HER2-targeting ADC system has not yet been developed for clinical use.

ADCs contain antibodies, linkers, and small molecule drugs. Antibodies selectively recognize antigens preferentially expressed on or near tumor cells [6] and bind to specific epitopes. Potent cytotoxic drugs effectively exert their cytotoxic effects through mechanisms such as cell signaling, cell cycle arrest, apoptosis and necrosis [7–10]. Linkers, which contain bifunctional groups, conjugate to the antibody on one end and to the drug on the other end [11, 12].

ADCs aim to take advantage of antibody specificity to selectively deliver potent cytotoxic drugs to antigen-expressing cancer cells. To fully realize the goal of targeted therapy with improved efficacy and tolerability, each component of the ADC should be optimized and various parameters should also be considered, such as selection of an appropriate antigen target and conjugation method [5]. Therefore, the efficacy depends on whether the combination of these parts is well matched.

Conjugation methods are divided into two categories, the cleavable system and the noncleavable system, according to the linker. The former system allows the drug to be released from the antibody after entering the cell, but the latter cannot be separated. Commonly used linkers are cleavable valine-citrulline (VC) [13, 14] and noncleavable N-succinimidyl 4-(maleimidomethyl) cyclohexane-carboxylate (SMCC) [15, 16].

Currently, VC containing a maleimide group with MMAE (a potent cytotoxic drug) is classified in one class; SMCC containing an NHS-ester group with DM1 (another potent cytotoxic drug) is classified in another class. VCMMAE is usually attached to the side chain of Cys, whereas SMCC-DM1 is usually connected to a Lys residue of the antibody. To prepare the ADCs, the former needs interruption of the interchain disulfide bonds, but the latter does not.

Human epidermal growth factor receptor 2 (HER2) is an important target membrane protein for cancer treatment and is a member of the EGFR family of transmembrane receptors [17]. It is overexpressed in a broad number of cancers [18–22]. In particular, amplification and overexpression of HER2 occurs in 25% to 30% of human breast cancer cases and is associated with a poor prognosis [23, 24].

Several HER2-targeting therapies, such as lapatinib (a small molecule) [25], trastuzumab [26–28], pertuzumab [29], and T-DM1 (ado-trastuzumab emtansine, Kadcyla^®^) [16], have been approved for patients with HER2-positive tumors. Although monoclonal antibodies (mAbs) are used to treat cancer patients, they need to be combined with chemotherapy, and many other mAbs have shown insufficient clinical activity [25, 30, 31]. Therefore, substantial effort has been devoted to empowering mAbs through various modifications, such as ADCs. To date, T-DM1 is the only clinical ADC among the HER2-targeting drugs, which employs SMCC connected to DM1, a tubulin polymerization inhibitor, in Herceptin^®^ [9].

Other than T-DM1, SGN-35 (brentuximab vedotin, Adcetris^®^), which has been approved by the FDA, has shown pronounced clinical activity at tolerated doses. It is indicated for the treatment of relapsed Hodgkin’s and systemic anaplastic large cell lymphomas that are characterized by high expression of the target antigen CD30 on the surface of malignant cells. SGN-35 employs VC-PABC to conjugate MMAE, a tubulin polymerization inhibitor, with brentuximab [13, 14]. It is worth noting that T-DM1 is a member of the noncleavable system, but SGN-35 is a member of the cleavable ADC system, and they target different antigens.

In this study, we developed an anti-HER2 ADC based on the humanized mouse antibody H32 [32]. Potent cytotoxic drugs, such as DM-1 and MMAE, were attached through cleavable or noncleavable linkers, and the efficiency of the conjugation procedures were evaluated by percent antibody recovery. Cytotoxic assays were performed to compare the *in vitro* potencies of our ADCs to the commercially available T-DM1.

## Materials and methods

### Chemicals and reagents

Ammonium sulfate, sodium phosphate, sodium chloride, tris(2-carboxyethyl) phosphine (TCEP), N-acetylcysteine (NAC), isopropyl alcohol (IPA), dimethyl sulfoxide (DMSO), 2-mercaptoethanol (2-ME), ethylenediaminetetraacetic acid (EDTA), phosphate-buffered saline (PBS), and TWEEN^®^ 20 were purchased from Sigma-Aldrich. Centrifugal filter tubes (Amicon-30 kDa) were purchased from Merck Millipore. N-Succinimidyl-4-(maleimidomethyl) cyclohexanecarboxylate-emtansine (SMCC-DM1) was obtained from ALB Technology. Kadcyla^®^ was purchased from Roche. Maleimidocaproyl-valine-citrulline-monomethyl auristatin E (VCMMAE) was obtained from MedChem Express. Lithium dodecyl sulfate (LDS) sample loading buffer (4X) and 12% acrylamide PAGE gel were purchased from Invitrogen. NUNC 96-well Maxisorb immunoplates were purchased from Thermo Fisher Scientific.

### Preparation of H32-VCMMAE

H32, a new anti-HER2 antibody, was humanized from the mouse antibody, which bound to a novel epitope on domain I of HER2/ECD and caused internalization and depletion of the HER2 receptor on HER2-overexpressing cells [32, 33]. For humanization, the complementarity-determining regions (CDRs) of the VL and VH domains were grafted into the human germline IGKV1-NL1*01/IGHV3-23*04 framework as previously described [33, 34]. The humanized variant was based on the oligonucleotide-directed mutagenesis procedure described previously [35].

In brief, six phosphorylated oligonucleotides for CDR swap were annealed to a uracilated single-stranded DNA template of V3c-HC TAA [32] at a molar ratio of 3:1 (oligonucleotide:ssDNA) by heating the mixture at 90°C for 2 minutes, followed by a temperature decrease of 1°C/min to 20°C in a thermal cycler. Subsequently, the template-primer annealing mixture was incubated in 0.32 mM ATP, 0.8 mM dNTPs, 5 mM DTT, 600 units of T4 DNA ligase, and 75 units of T7 DNA polymerase (New England Biolabs) to prime *in vitro* DNA synthesis. After overnight incubation at 20°C, the synthesized dsDNA was transformed into *Escherichia coli* ER2738 at 2500 V with an electroporator. A single colony was randomly selected and placed into a deep 96-well culture plate, and its secreted scFvs were induced with 1 mM IPTG. After overnight incubation at 37°C with vigorous shaking, the cultures were centrifuged at 3000 g for 10 minutes at 4°C. Then, the supernatants were employed for ELISA binding assay against the HER/ECD antigen [32, 33]. The clones with high ELISA signal intensity (OD_450_ > 2.0) were picked and subjected to DNA sequencing. The resulting humanized H32 scFv was then converted into IgG format, and its HER2/ECD binding affinity and epitope were further validated [32, 33].

### Preparation of H32-MCC-DM1 (H32-DM1)

DM1 with SMCC was conjugated to the H32 monoclonal antibody to form H32-MCC-DM1 (H32-DM1) by amide bond coupling through the activated NHS ester. The pH 7.25 conjugation buffer containing 100 mM sodium phosphate and 150 mM sodium chloride was first prepared to make an appropriate environment for the synthesis. The concentration of H32 antibody was adjusted to 13.5 nmol/ml (2 mg/mL, 1mL) by dilution with the conjugation buffer. SMCC-DM1 (217.2 μg, 202.5 nmol, 15 equiv) was dissolved in DMSO (21.72 μL) and added to the stock H32 stock solution (2 mg, 13.5 nmol, 1 equiv). The mixture was incubated at 32°C for 2, 4, 6 and 24 hours to afford different DAR and drug distributions. Unreacted SMCC-DM1 and side products (N-hydroxysuccinimide) were removed by ultrafiltration (Amicon-30 kDa). The H32-DM1 series products were stored at 4°C for later analysis and application.

### Preparation of H32-VCMMAE

MMAE with maleimide-modified VC was conjugated to the H32 monoclonal antibody by Michael addition to form H32-VCMMAE. The pH 7.5 conjugation buffer solution contained 50 mM sodium phosphate, 50 mM sodium chloride and 2 mM EDTA. The H32 stock solution was diluted to a concentration of 13.5 μM (2 mg/mL) with conjugation buffer. The disulfide bridges were then reduced with various molar equivalents of TCEP (1.8-fold, 5.4-fold or 10.8-fold) at 30°C for 2 hours to afford different numbers of available sulfhydryl groups on the antibody molecule.

The reduced antibody was then conjugated with 5.4, 10.8, or 21.6 equivalents of VCMMAE (with respect to the molarity of whole antibody molecules) dissolved in DMSO at 30°C to afford a range of different DARs. The reactions were allowed to proceed for 1.5, 3, or 20 hours. Upon completion, N-acetylcysteine (NAC) was employed to quench the reaction, and the unreacted VCMMAE and side products were removed by ultrafiltration using Amicon-30 kDa. The resulting H32-VCMMAE ADCs were stored at 4°C for later analysis and application.

### ESI-Q-TOF MS analysis

The ADC sample was diluted to a concentration of 0.1 mg/ml with 0.1% formic acid for intact mass analysis. For monoclonal intact antibody analysis, separation was performed using a Waters UPLC System on a Waters ACQUITY UPLC column (Waters, C4 BEH300, 1.7 μm, 2.1 mm × 50 mm) coupled with a TripleTOF^™^ 5600 System. The mobile phase consisted of solvent A: 0.1% aqueous formic acid (FA) and solvent B: acetonitrile (ACN) with 0.1% formic acid. The optimal flow rate and column temperature were determined experimentally to be 200 μl/min and 80°C. The determination of the intact mass, including spectral deconvolution and mass reconstruction, was performed by BioPharmaView^™^ software.

### Analysis of DAR distribution by hydrophobic interaction chromatography

The DAR distributions of the ADCs were analyzed by hydrophobic interaction chromatography (HIC) with a TSKgel Butyl-NPR column (2.5 μm particles and 4.6 mm ID × 3.5 cm, Tosoh Bioscience, Montgomeryville, PA).

Mobile phase A, an aqueous solution of 1.8 M ammonium sulfate and 25 mM sodium phosphate at pH 7, and mobile phase B, a mixture of 75% (v/v) 25 mM sodium phosphate aqueous solution at pH 7 and 25% (v/v) isopropyl alcohol, were used to elute the samples. A linear gradient from 100% mobile phase A to 100% mobile phase B over 12 minutes at a flow rate of 1 mL/min was employed to elute the samples. The temperature was set at 25°C. Peaks were monitored by their absorbance at 248 nm.

Finally, the drug-to-antibody ratio (DAR) of the samples was calculated by

$$\text{DAR}=\sum \text{n}\times {\text{A}}_{\text{n}}/\sum {\text{A}}_{\text{n}}$$ (1)

where n denotes the number of drugs attached to the antibody (DAR species) and A_n_ denotes the area under each DAR species peak cluster.

### Quantification and recovery (%) of the proteins

The protein contents of the samples were quantified by the standard Bradford method (Bio-Rad protein assay kit). BSA was employed to construct a calibration curve. The percent antibody recovery after the conjugation and clean-up procedures was defined as:

$$\text{Recovery}\;(\%)={\text{W}}_{\text{C}}/{\text{W}}_{\text{H}32}\times 100\%$$ (2)

where W_H32_ and W_C_ are the weights of H32 and the conjugated product, respectively.

### LDS-PAGE analysis of ADCs

Both reducing and nonreducing LDS-PAGE were conducted for H32, H32-DM1, and H32-VCMMAE. Lithium dodecyl sulfate (LDS) sample loading buffer (4X) and 1X working solution containing 106 mM Tris HCl, 141 mM Tris base, 2% LDS, 10% glycerol, 0.51 mM EDTA, 0.22 mM G250 Coomassie Blue, 0.175 mM phenol red, pH 8.5 with and without 2-mercaptoethanol (2-ME) were employed to prepare the samples. Samples (approximately 4 μg of protein per sample) were denatured at 95°C for 15 minutes. The mixtures were loaded on a 12% acrylamide PAGE gel for separation at 150 V/160 mA for approximately 1 hour.

After electrophoresis, the gels were stained with InstantBlue^™^ solution (Bio-Rad, Hercules, CA, USA). The gels were then destained overnight in ultrapure water. Gel images were captured by an HP Scanjet 8300.

### Binding affinities of the ADCs determined by ELISA

The half-maximal effective concentrations (EC_50_ values) of H32, H32-DM1, and H32-VCMMAE were determined by titrations of IgG antibodies on immobilized HER2/ECD with ELISA. In brief, the HER2/ECD antigen (0.3 μg per well) in PBS buffer (pH 7.4) was coated on NUNC 96-well Maxisorb immunoplates for 16 hours at 4°C and then blocked with 5% skim milk in PBST (phosphate-buffered saline with Tween-20) for 1 hour. In the meantime, samples in PBST with 5% milk were prepared at 11 concentrations by performing two-fold serial dilutions.

After blocking, 100 μl of each diluted sample was added to each well and incubated for 1 hour with gentle shaking. The plate was washed 4 times with 300 μl of PBST, and then 100 μl of horseradish peroxidase/anti-human IgG antibody conjugate (2000× dilution) in PBST with 5% milk was added for incubation for 1 hour at room temperature. After washing 4 times with PBST buffer and twice with PBS, the samples were treated with 3,3’,5,5’-tetramethylbenzidine peroxidase substrate for 3 minutes, quenched with 1.0 M HCl and measured at 450 nm by an ELISA reader. The EC_50_ (ng/ml) was calculated by ED_50_ Plus v1.0 software.

### IC_50_ determination from the cytotoxicity assay

N87, SK-BR-3, and BT474 cells were obtained from the American Type Culture Collection (ATCC). N87 and SK-BR-3 cells were cultured in RPMI 1640 (Gibco) with 10% fetal bovine serum (Gibco) and penicillin-streptomycin (100×; Gibco). BT474 cells were cultured in hybrid-Care medium (ATCC) with 10% fetal bovine serum (Gibco) and penicillin/streptomycin/glutamine (100×; Gibco). Approximately 10^4^ cells were seeded in 96-well plates for each well.

To determine the IC_50_ values of the ADCs (both the H32-DM1 and H32-VCMMAE series), different concentrations of each conjugate was directly added to the culture medium. After the mixture was incubated at 37°C for 16 hours, the culture medium was replaced with fresh medium. For the H32 control, the same treatment procedure as that of the ADCs was followed. The 16 hours short pulse of ADCs and then 3 day’s culture focused on cytotoxicity through ADCs-mediated endocytosis and minimize cytotoxicity through non-specific pinocytosis of ADCs by cancer cells [36]. After the cells were cultured for 3 days at 37°C, the number of viable cells was quantified by using the WST-1 (Roche) reagent, and the absorbance was measured at OD_450_. The percentage of cell viability was calculated by

$$\text{Cell}\;\text{viability}\;(\%)=[{}^{\text{T}}{\text{OD}}_{450\;\text{nm}}{/}^{\text{C}}{\text{OD}}_{450\;\text{nm}}]\times 100\%$$ (3)

where T are the cells treated with H32, H32-DM1, and H32-VCMMAE and C is the untreated control cells. The IC_50_ value (nM) was calculated by ED_50_ Plus v1.0 software.

### Flow cytometry

Gastric cancer cell line N87, breast cancer cell line SKBR-3 and BT474 cells (3×10^5^) were suspended in 96-well plates and stained with 1 μg/mL anti-HER2 H32 IgG1 and the same concentration of control antibody. After staining, the cells were washed twice with PBS and then incubated with Alexa Fluor 633-labeled antibody against human IgG (Invitrogen) for 30 minutes at 4°C. The fluorescence intensity was analyzed using a BD FACS Canto II (BD Biosciences).

## Results

### Preparation of H32-DM1 and H32-VCMMAE ADCs

**Fig 1:**
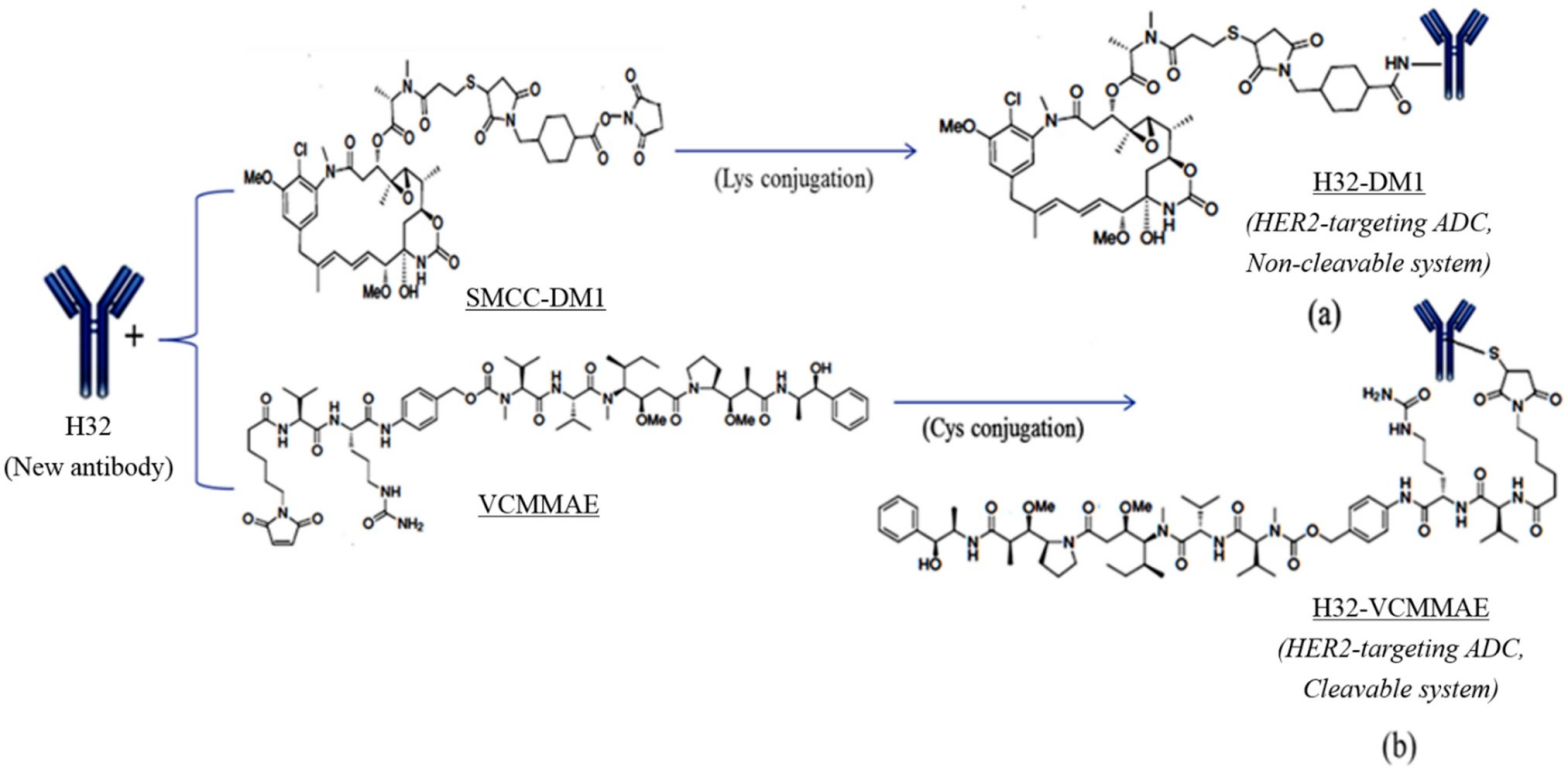
Illustration of (a) H32-DM1 and (b) H32-VCMMAE preparations. SMCC-DM1 and VCMMAE were conjugated to H32 by an SN2 reaction and Michael addition to form H32-DM1 and H32-VCMMAE, respectively.

In this study, antibody drug conjugates (ADCs) with different properties were prepared by different conjugation chemistry. We focused on the HER2 antigen target and investigated the two ADC systems, including a noncleavable H32-DM1 ADC and a cleavable H32-VCMMAE ADC, to compare different effects, such as recovery, DAR and cytotoxicity. DM1 and VCMMAE are covalently bound to a Lys residue by amide coupling through an activated ester and Michael addition through a Cys residue of H32 to form H32-DM1 (Fig 1a) and H32-VCMMAE (Fig 1b) as the ADCs, respectively.

### Characterization of the DAR and drug distribution of various ADCs

The selection of the most appropriate method for a specific ADC is dependent on the properties of the linker, the properties of the drug and the choice of attachment site [37]. To analyze the properties of H32-DM1 and H32-VCMMAE, ESI-Q-TOF MS and HIC-HPLC methods were employed. Our data show that the former method is suitable for H32-DM1, and the latter method is suitable for H32-VCMMAE to analyze the DAR and drug distribution.

### ESI-Q-TOF MS analysis

**Fig 2:**
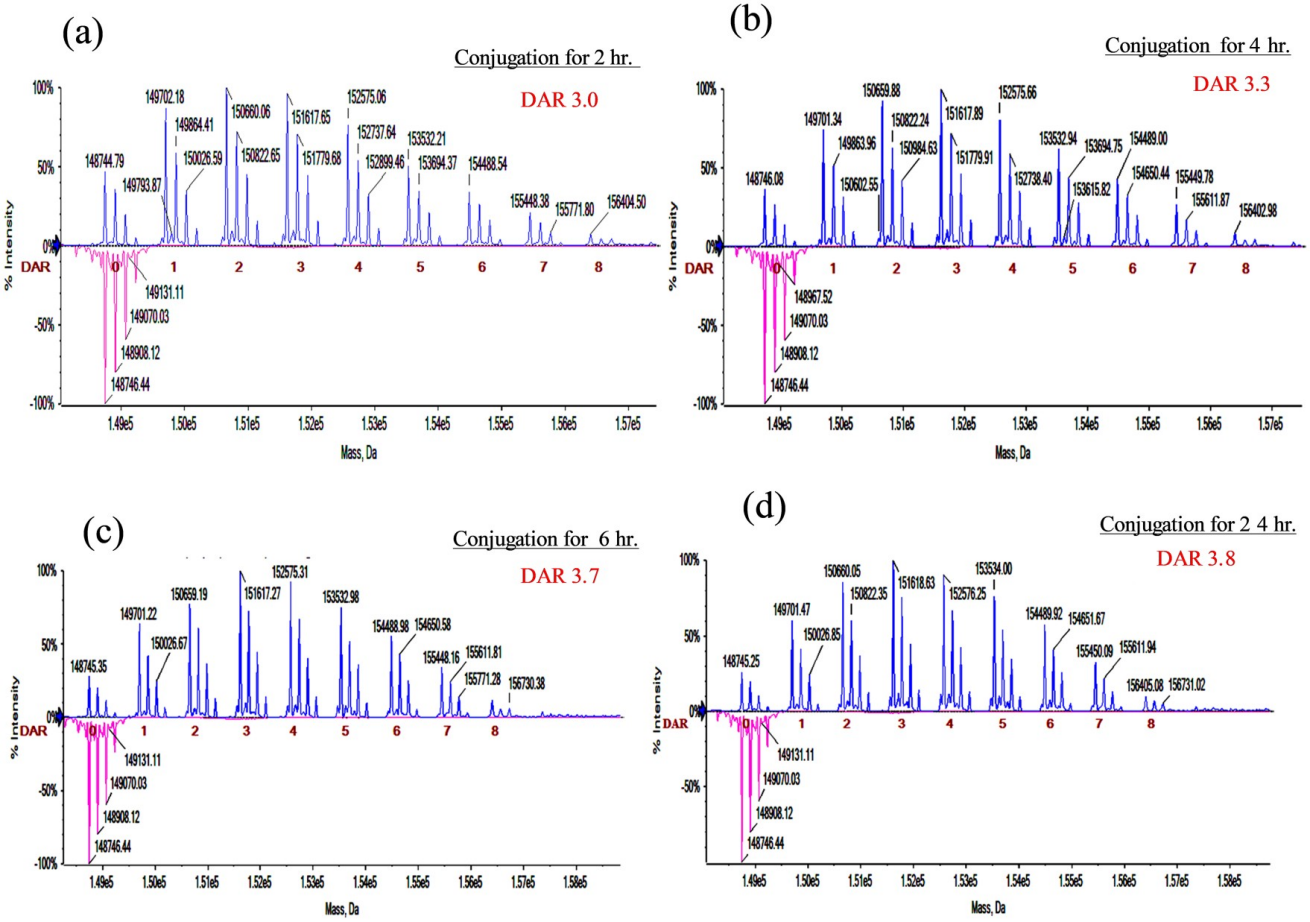
Representative ESI-Q-TOF MS spectra of the H32-DM1 series. (a) DAR 3.0 and (b) DAR 3.3, the main distributed payloads of which were 1, 2, 3, and 4; (c) DAR 3.7 and (d) DAR 3.8, the main distributed payloads of which were 2, 3, 4, and 5.

ESI-Q-TOF MS was employed to analyze H32-DM1 conjugated with different DARs and drug distributions. Fig 2a and 2b show that after reaction for 2 hours or 4 hours, the significant loading distributions of DM1 in H32 were 1, 2, 3, and 4 with average DARs of 3.0 (denoted H32-DM1_3.0) and 3.3 (denoted H32-DM1_3.3), respectively. Likewise, Fig 2c and 2d show that after reaction for 6 hours or 24 hours, the main distribution of DM1 in H32 shifted to 2, 3, 4, and 5 with average DARs of 3.7 (H32-DM1_3.7) and 3.8 (H32-DM1_3.8), respectively.

The results show that the average DAR increases as the reaction time increases. However, there is no significant difference in the average DAR between the 6-hour and 24-hour reactions, suggesting a saturation effect. When the DAR was above 3.7, the available active sites of H32 could have almost all been conjugated to DM1, so that the average DAR cannot be significantly further increased, despite the reaction time being increased from 6 hours to 24 hours.

### HIC-HPLC analysis

**Fig 3:**
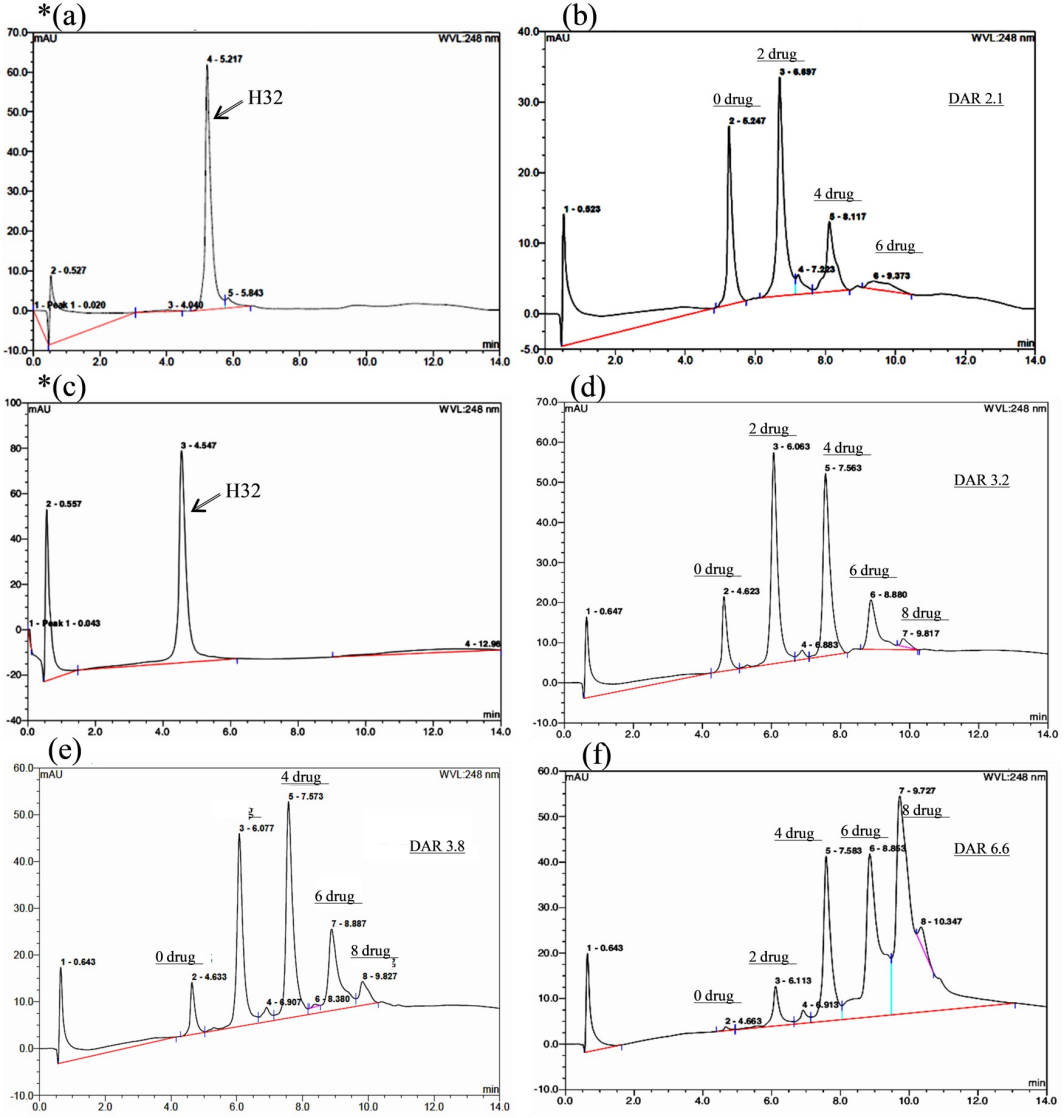
Representative HIC-HPLC chromatograms of the H32-VCMMAE series. (a, c) H32 and (b, d, e, f) H32-VCMMAE with different drug to antibody ratios (DARs) and drug distributions. (b) DAR 2.1, the main distributed payload of which was 2 against (a); (d) DAR 3.2 and (e) DAR 3.8, the main distributed payloads of which were 2 and 4 against (c); (f) DAR 6.6, the main distributed payloads of which were 4, 6 and 8 against (c).

H32-VCMMAE conjugated with different DARs was analyzed by HIC-HPLC. This ADC was constructed by reducing the four interchain disulfide bonds of H32 and possessed a different distribution of drug payloads, such as 2, 4, 6 and 8, as shown in Fig 3. Unconjugated H32 eluted at 5.22 minutes (Fig 3a) as a single peak. In Fig 3b, the absorbance profile of VCMMAE-conjugated H32 exhibits several well-resolved peaks with different retention times at 5.25, 6.70, 8.12 and 9.37 minutes, which indicate that the drug payloads of H32-VCMMAE are 0, 2, 4 and 6.

In addition, the major drug payload of VCMMAE in H32 is 2, with an average DAR of 2.1 (denoted H32-VCMMAE_2.1) (Fig 3b). H32-VCMMAE_2.1 was prepared by Michael addition in which H32 reacted with 5.4 equivalents of VCMMAE for 20 hours after the interchain disulfide bonds of H32 were reduced by TCEP (1.8-fold) for 2 hours.

In Fig 3d and 3e, the absorbance profile of various VCMMAE-conjugated H32 molecules also exhibited well-resolved peaks with different retention times at 4.6, 6.1, 7.6, 8.9 and 9.8 minutes, which indicated that the drug payloads of H32-VCMMAE were 0, 2, 4, 6 and 8 in H32 (Fig 3c). The major drug payloads of VCMMAE in H32 were 2 and 4, with average DARs of 3.2 (denoted H32-VCMMAE_3.2) (Fig 3d) and 3.8 (denoted H32-VCMMAE_3.8) (Fig 3e).

In Fig 3f, the absorbance profile of VCMMAE-conjugated H32 displays well-resolved peaks shifted toward higher retention times, suggesting drug payloads of 0, 2, 4, 6 and 8 against H32 (Fig 3f). The average DAR was 6.6 (denoted H32-VCMMAE_6.6). The results indicate that H32 could completely react with VCMMAE, with a significantly increased DAR.

### Quantification of protein recovery after conjugation

The recovery rate indicates whether the precipitation of the antibody in different reaction processes is prone to take place or not. In our studies, the recovery rates of various H32-DM1 ADCs were approximately 60% to 70%, but H32-VCMMAE_3.2, H32-VCMMAE_3.8, and H32-VCMMAE_6.6 were approximately 90%, as shown in Table 1.

**Table 1:** Recovery (%) of the H32-DM1 series and H32-VCMMAE series.

ADC_DAR	Wc (mg)	Recovery (%)	ADC_DAR	Wc (mg)	Recovery (%)
H32-DM1_3.0	1.41	70.67	*H32-VCMMAE_2.1	1.06	52.99
H32-DM1_3.3	1.26	62.82	H32-VCMMAE_3.2	1.83	91.65
H32-DM1_3.7	1.50	74.90	H32-VCMMAE_3.8	1.82	90.80
H32-DM1_3.8	1.39	69.46	H32-VCMMAE_6.6	1.81	90.45

WC is the weight of the conjugated product.

*The reaction time was controlled for 20 hours when VCMMAE (5.4 equivalents) conjugated to H32 had been reduced by TCEP (1.8-fold) for 2 hours.

In the H32-DM1 reaction process, although H32 was subjected to different reaction times from two hours to 24 hours, their precipitation showed no significant differences. In the preparation of H32-VCMMAE, H32 precipitated to a greater extent. However, the problem of precipitation can be overcome by shortening the conjugation reaction time.

### LDS-PAGE assay

**Fig 4:**
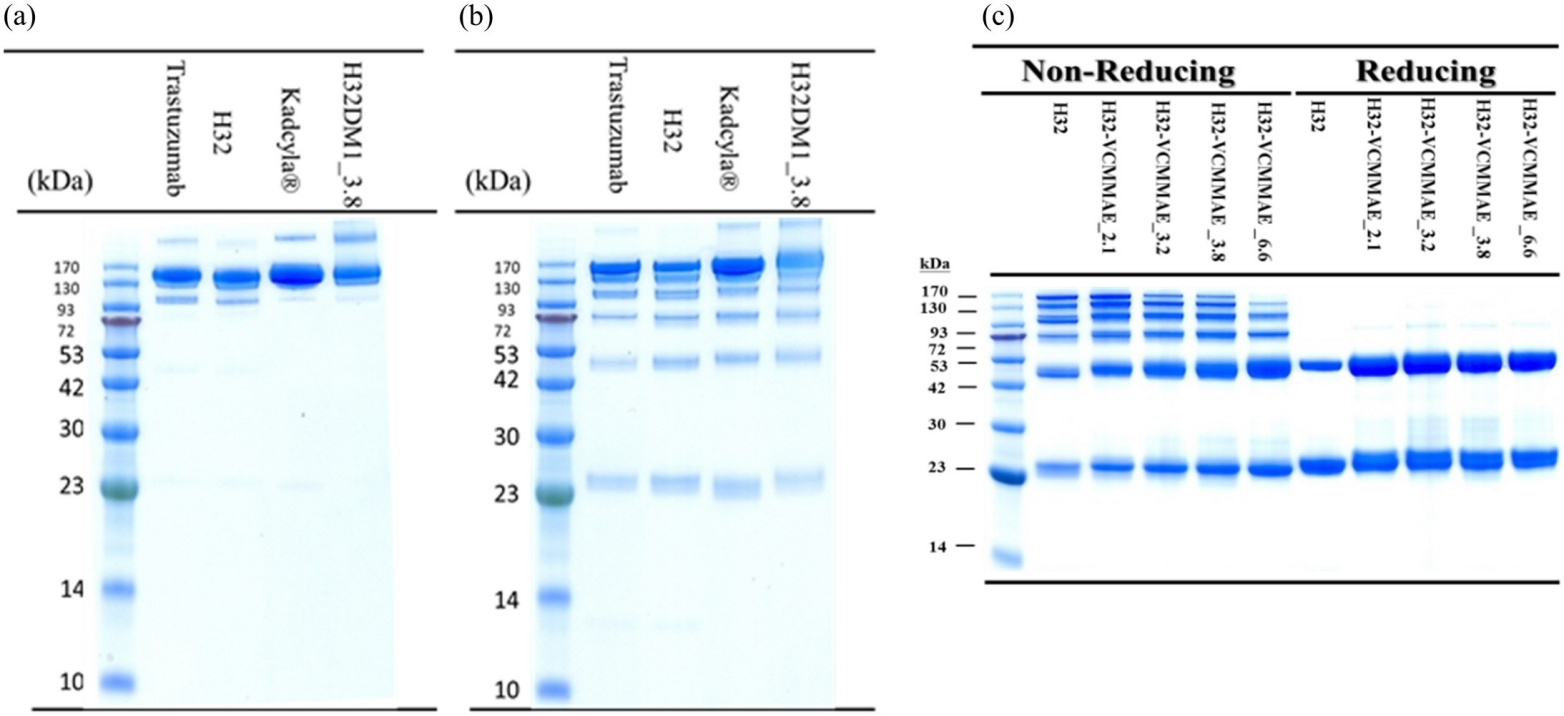
LDS-PAGE images of trastuzumab, H32, Kadcyla^®^, H32-DM1_3.8 and H32-VCMMAE. (a) Samples were mixed with LDS loading buffer and placed at 25°C for 15 minutes. (b, c) The samples were mixed with LDS loading buffer and heated at 95°C for 15 minutes.

Antibodies are composed of two heavy chains and two light chains linked by four disulfide bonds. The difference in the molecular weight of H32 before and after conjugation with the small cytotoxic agent are displayed in Fig 4.

Fig 4a and 4b show the LDS-PAGE images of trastuzumab, H32, Kadcyla^®^, and H32-DM1_3.8. When samples were mixed with LDS loading buffer and placed at 25°C before running the gel, the main band was at ~150 kDa (Fig 4a). The results indicated that the antibodies were intact with two heavy chains and two light chains. Furthermore, their molecular weights were in the order of Kadcyla^®^ > Trastuzumab and H32-DM1_3.8 > H32, which supported that the antibodies conjugated with DM1 successfully. As the samples were mixed with LDS loading buffer and heated to 95°C before running the gel, the bands showed six molecular weights at approximately 25, 50, 75, 100, 125 and 150 kDa (Fig 4b). These results suggested that some antibodies were no longer folded properly due to heating. It is worth noting that the 3D structure of an antibody requires not only disulfide bonds but also noncovalent bonds such as hydrogen bonds and hydrophobic bonds.

Fig 4c shows images of H32-VCMMAE under nonreducing and reducing conditions. The nonreducing part displayed two main bands at 25 kDa and 50 kDa and four other light bands, which suggested that some disulfide bonds of the antibody were slightly reduced in the H32-VCMMAE reaction process. When the 2-ME samples were mixed with LDS loading buffer and heated to 95°C before running the gel, two single bands (heavy and light chains) were observed. The images also show that the molecular weights increased with increasing DAR.

### Antigen binding affinities of H32, H32-DM1 and H32-VCMMAE

The binding affinities (EC_50_ values) of conjugated H32 with the small cytotoxic agent before and after conjugation were compared. The EC_50_ values and the graded dose-response curves were calculated as shown in Table 2 and S1 Fig. The EC_50_ value of H32 is approximately 1.944 ± 0.095 ng/ml, indicating good affinity. The EC_50_ values of H32-DM1_3.0, H32-DM1_3.3, H32-DM1_3.7 and H32-DM1_3.8 were approximately 4.294 ± 0.429 ng/ml, 4.121 ± 0.496 ng/ml, 4.477 ± 0.162 ng/ml and 3.69 ± 0.349 ng/ml, respectively, which indicates no significant difference among their binding affinities.

**Table 2:** EC_50_ values of the H32-DM1 series and H32-VCMMAE series.

ADC_DAR	EC_50_ (ng/ml)	SD (n = 4)	ADC_DAR	EC_50_ (ng/ml)	SD (n = 3)
H32	1.944	0.095	H32	1.308	0.032
H32-DM1_3.0	4.294	0.429	H32-VCMMAE_2.1	1.426	0.572
H32-DM1_3.3	4.121	0.496	H32-VCMMAE_3.2	2.018	0.710
H32-DM1_3.7	4.477	0.162	H32-VCMMAE_3.8	1.951	0.520
H32-DM1_3.8	3.690	0.349	H32-VCMMAE_6.6	3.829	0.897

The values were calculated as described in the Materials and Methods section.

These EC_50_ values were slightly higher than that of H32, which suggested that their binding affinities decreased slightly when H32 was conjugated to DM1. The EC_50_ values of H32-VCMMAE_2.1, H32-VCMMAE_3.2, H32-VCMMAE_3.8 and H32-VCMMAE_6.6 were approximately 1.426 ± 0.572 ng/ml, 2.018 ± 0.710 ng/ml, 1.951 ± 0.520 ng/ml and 3.829 ± 0.897 ng/ml, respectively, which indicates that their binding affinities are similar, except for H32-VCMMAE_6.6.

The above results suggest that the overall antibody structure was not affected to a significant degree, so those ADCs still bind to the HER2 antigen effectively.

### H32, H32-DM1, and H32-VCMMAE cell viability assays

HER2 has become an increasingly important prognostic and predictive factor in breast cancer [38], and it is a new prognostic factor and a novel therapeutic target in gastric cancer [39]. In this study, two human breast cancer cell lines, SK-BR-3 and BT474, with high expression levels of the HER2 protein, and a gastric cancer cell line, N87, also overexpressing the HER2 antigen, were employed. To show the optimized combination of the ADC components, the IC_50_ (half maximal inhibitory concentration) was measured to determine their efficacies. The IC_50_ values and the graded dose cytotoxicity curve were calculated as shown in Table 3 and S2 Fig.

**Table 3:** IC_50_ values of the H32-DM1 series and H32-VCMMAE series.

	N87	SK-BR-3	BT474
ADC_DAR	IC_50_ (nM) ± SD (n = 3)		
H32	—	—	—
H32-DM1_3.0	0.84 ± 0.15	0.08 ± 0.01	0.77 ± 0.15
H32-DM1_3.3	0.65 ±0.04	0.05 ± 0.02	0.65 ± 0.05
H32-DM1_3.7	0.75 ± 0.06	0.06 ± 0.04	0.79 ± 0.24
H32-DM1_3.8	0.76 ± 0.15	0.05 ± 0.05	0.54 ± 0.07
Kadcyla	1.57 ± 0.10	0.24 ± 0.12	5.89 ± 3.51
H32	—	—	—
H32-VCMMAE_2.1	0.78 ± 0.02	0.07 ± 0.01	0.11 ± 0.02
H32-VCMMAE_3.2	0.10 ± 0.21	0.05 ± 0.01	0.08 ± 0.02
H32-VCMMAE_3.8	0.62 ± 0.08	0.04 ± 0.00	0.06 ± 0.01
H32-VCMMAE_6.6	0.50 ± 0.04	0.03 ± 0.01	0.02 ± 0.00

Samples were incubated with N87, SK-BR-3 and BT474 cells. The values were calculated as described in the Materials and Methods section.

The relative viabilities of N87 cells treated with H32-DM1 and H32-VCMMAE were lower than that of Kadcyla^®^; in other words, their efficacy to kill cancer cells is higher than that of Kadcyla^®^. However, H32 alone had no efficacy in this experiment (S2a and S2b Fig). In the H32-DM1 and H32-VCMMAE series, the IC_50_ values were approximately 0.6 ~ 0.9 nM and 0.5 ~ 0.8 nM, respectively, with cytotoxicities twice that of Kadcyla^®^. Among the series, H32-VCMMAE_6.6 had the best cytotoxicity, with an IC_50_ value of 0.50 ± 0.04 nM (Table 3).

The relative viabilities of SK-BR-3 and BT474 cells for both the H32-DM1 and H32-VCMMAE series were lower than that of Kadcyla^®^ (S2c, S2d, S2e and S2f Fig). In SK-BR-3 cells, the IC_50_ values of the H32-DM1 series were approximately 0.05 ~ 0.08 nM, indicating that their cytotoxicity efficacy is approximately 3 ~ 5 times better than that of Kadcyla^®^. For the H32-VCMMAE series, their IC_50_ values were approximately 0.03 ~ 0.07 nM. Among them, the efficacy of H32-VCMMAE_6.6 was the cancer cell killer; its IC_50_ value was 0.03 ± 0.01 nM.

In BT474 cells, the IC_50_ values of the H32-DM1 series were approximately 0.5 to 0.8 nM, which is approximately 8 to 10 times lower than that of Kadcyla^®^. The IC_50_ values of the H32-VCMMAE series were approximately 0.02 ~ 0.1 nM. Among them, H32-VCMMAE_6.6 is again the best, with an IC_50_ value of 0.02 ± 0.00 nM. Overall, their cytotoxicity was in the order of H32-VCMMAE series > H32-DM1 series > Kadcyla^®^.

## Discussion

Most of the ADCs currently on the market and in clinical development carry tubulin polymerization inhibitors such as T-DM1 and SGN-35 [40]. T-DM1- and SGN-35-containing maytansinoids and auristatins have displayed a good therapeutic index [13–16]. The optimized combination of ADCs could be an important factor for the development of ADCs and their anticancer performance.

In this regard, we employed H32 as a new antibody carrier to develop an optimal anti-HER2 ADC. A maytansinoid (DMI) and an auristatin (VCMMAE) were conjugated to H32. Amide bond formation through an activated ester and Michael addition were employed in various conditions to create a series of noncleavable H32-DM1 ADCs and cleavable H32-VCMMAE ADCs.

Cytotoxicity efficacy (see the IC_50_ values in Table 3) measures the cancer cell killing activity. Although the N87, SK-BR-3, and BT-474 cells are all HER2-overexpressing cell lines, their origins and properties are different. N87 cells, a gastric carcinoma cell line, are derived from the liver (metastatic site). SK-BR-3 cells, an adenocarcinoma (mammary gland/breast cancer) cell line, are derived from a pleural effusion (metastatic site). BT-474 cells are derived from solids, the primary origin of the cells [41]. In addition, HER2 expression at the cell surface level was in the order of BT-474 > SK-BR-3 according to the research results of Kute et al. [42]. In our study, the expression levels of HER2 were in the similar order of BT-474 > N87 > SK-BR-3 as analyzed by flow cytometry and shown in S3 Fig. It should be noted, however, that the susceptibility of cancer cells to HER2-targeting ADCs is not solely determined by the expression level of HER2 on the cell surface. Factors such as the rate of internalization and the efficiency of payload release also impact the potencies of ADCs. Our results of BT474 being less susceptible to the HER2-targeting ADC than SK-BR-3 were consistent with the literature [43]. Although H32 alone displays no cancer cell killing activity, H32 is an excellent carrier for developing ADCs because it has a superb cell penetrating properties. In our study, the cytotoxicity efficacies of ADCs against N87, SK-BR-3 and BT474 cells were in the order of H32-VCMMAE series > H32-DM1 series > Kadcyla^®^. The results indicate that in addition to DAR, optimizing the combination of ADC components is also crucial for killing cancer cells. We conclude that the optimized combination ADC was H32-VCMMAE_6.6.

## Supporting information

S1 Fig Antigen binding affinities of H32, H32-DM1 and H32-VCMMAE. EC_50_ values of the graded dose-response curve of (a) H32-DM1 and (b) H32-VCMMAE with various DARs.
(TIF)

S2 Fig The cancer cell killing activities of H32, H32-DM1, and H32-VCMMAE. IC_50_ values of the graded dose-response curve of (a, c, e) H32-DM1 and (b, d, f) H32-VCMMAE with various DARs. To assay the cancer cell killing activity of both series, the ADCs were incubated with various cancer cells, including (a, b) N87, (c, d) SK-BR-3 and (e, f) BT474.
(TIF)

S3 Fig HER2 expression varies among three different cancer cell lines. Binding of H32 to (A) gastric cancer cell line N87, (B) breast cancer cell line SKBR-3, and (C) breast cancer cell line BT474 was detected by immunofluorescent staining and flow cytometry. The histogram shows the florescence intensity of the cell only (black line), control IgG1 binding (blue line), and H32 binding (red line). The integrated values of H32 binding suggest that HER2 is expressed in the abundance order of BT474>N87>SKBR-3.
(TIF)

S1 Raw Images (PDF)

## Abbreviations

DAR: drug-to-antibody ratio
EC_50_: half-maximal effective concentration
ESI: electron spray ionization
HIC: hydrophobic interaction chromatography
IC_50_: half-maximal inhibitory concentration
LDS: lithium dodecyl sulfate
NHS: N-hydroxysuccinimide
PABC: p-aminobenzyloxycarbonyl
Q-TOF: quadruple time-of-flight
SGN-35: brentuximab vedotin, (Adcetris^®^)
SMCC: succinimidyl 4-(N-maleimidomethyl)cyclohexane-1-carboxylate
TCEP: tris(2-carboxyethyl)phosphine
T-DM1: ado-trastuzumab emtansine (Kadcyla^®^)
VCMMAE: valine-citrulline-monomethyl auristatin E
