# Supplementary figures and images for: Preparation and characterization of antibody-drug conjugates acting on HER2-positive cancer cells
Source: PLoS One. 2020 Sep 28;15(9):e0239813. doi: 10.1371/journal.pone.0239813 (PMC7521679; doi:10.1371/journal.pone.0239813)

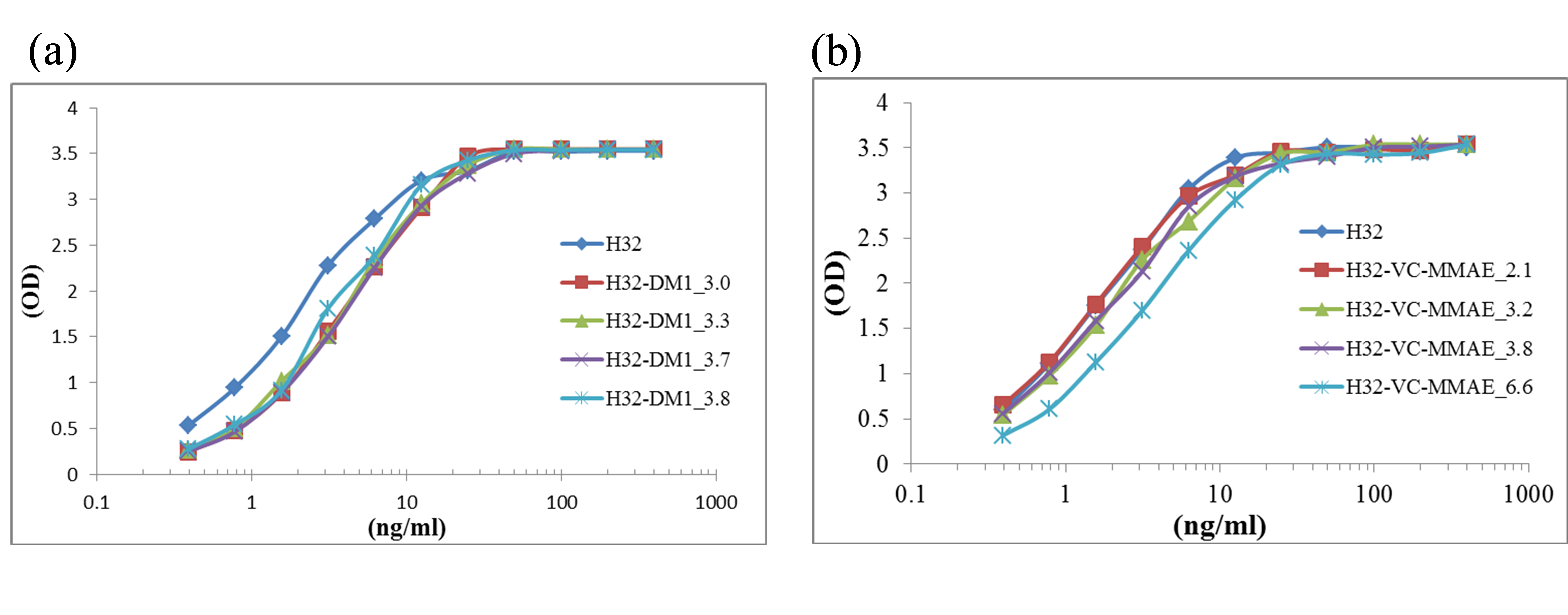

Supplement: S1 Fig — EC50 values of the graded dose-response curve of (a) H32-DM1 and (b) H32-VCMMAE with various DARs. (TIF) [file pone.0239813.s001.tif]

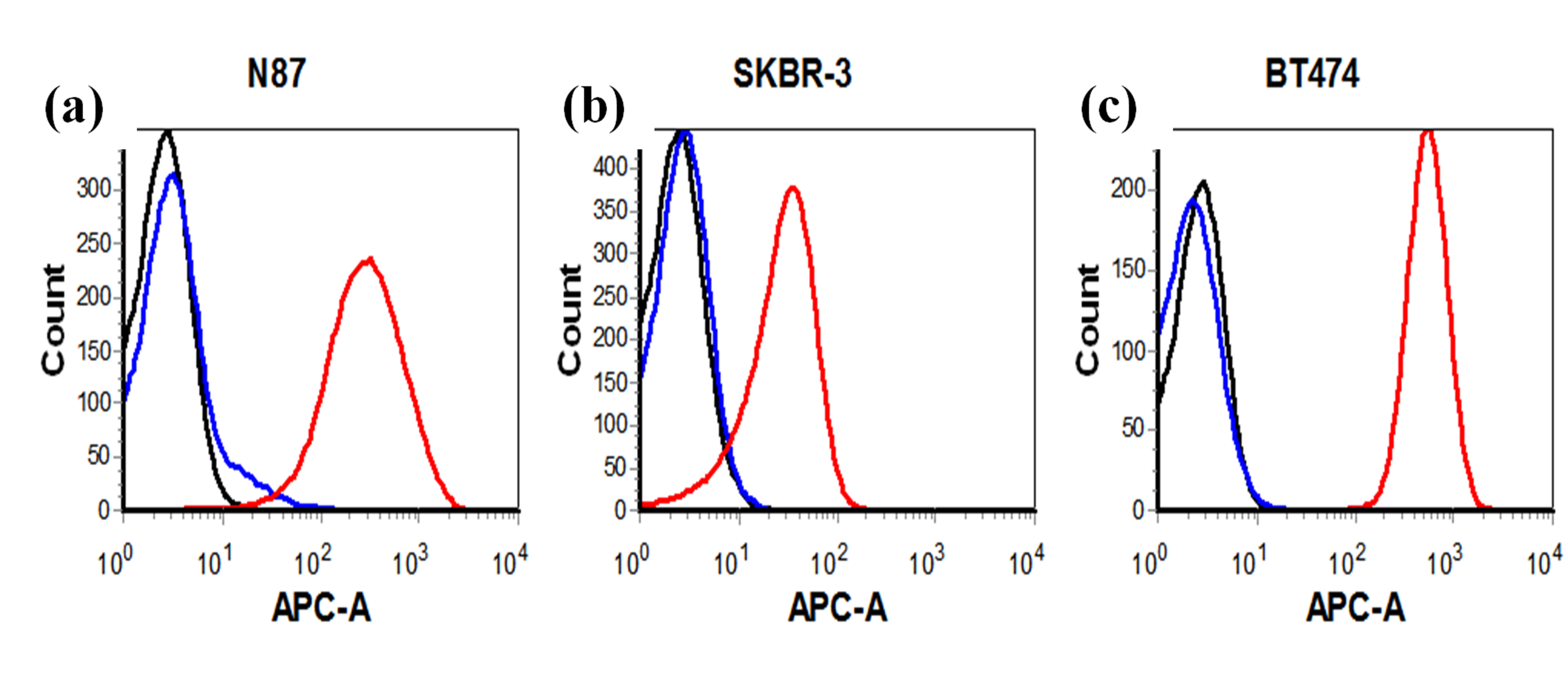

Supplement: S3 Fig — Binding of H32 to (A) gastric cancer cell line N87, (B) breast cancer cell line SKBR-3, and (C) breast cancer cell line BT474 was detected by immunofluorescent staining and flow cytometry. The histogram shows the florescence intensity of the cell only (black line), control IgG1 binding (blue line), and H32 binding (red line). The integrated values of H32 binding suggest that HER2 is expressed in the abundance order of BT474>N87>SKBR-3. (TIF) [file pone.0239813.s003.tif]

Fig 4a 4b

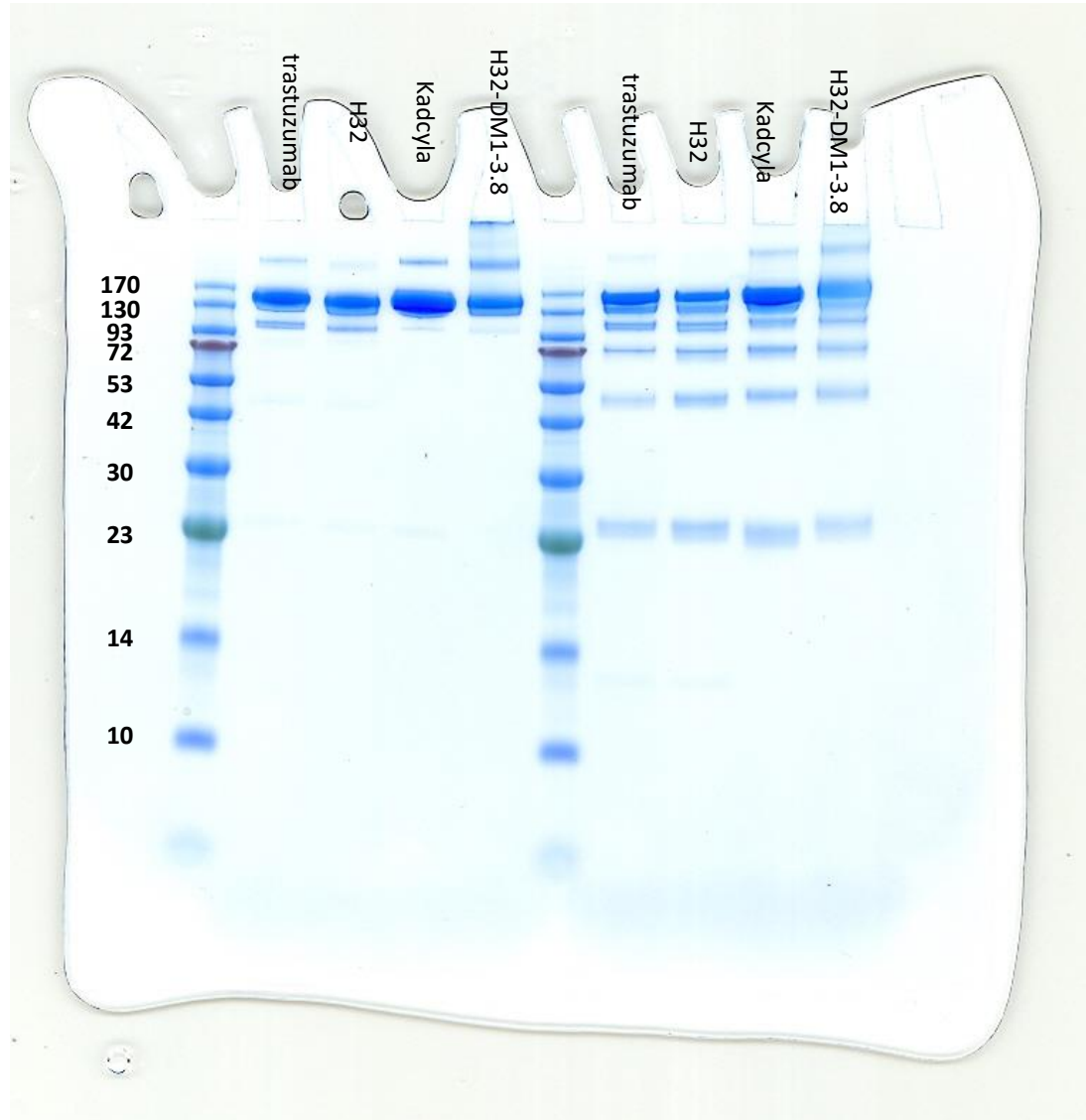

Fig 4c

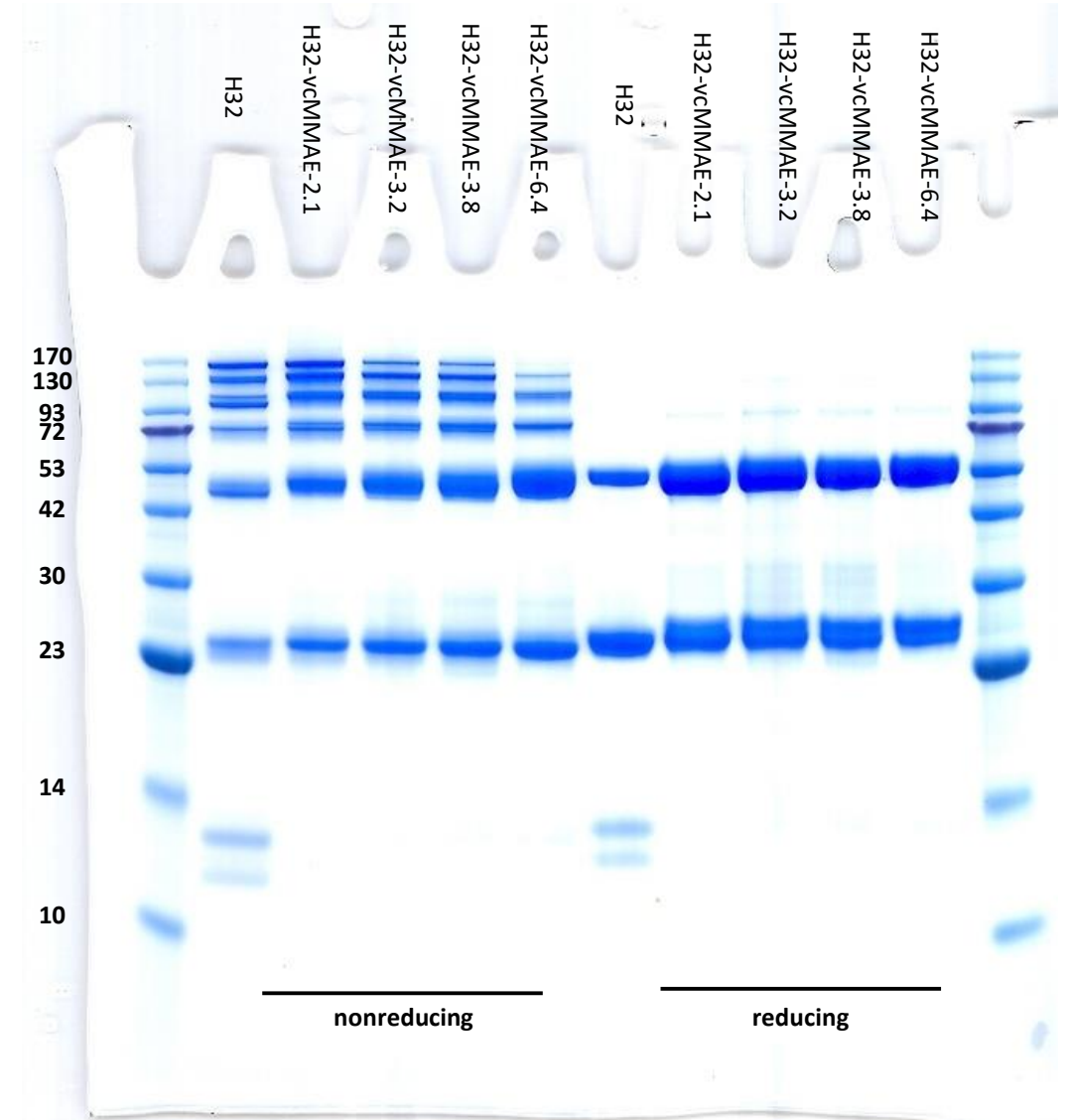

Supplement: S1 Raw Images — (PDF) [file pone.0239813.s004.pdf]
